# Sublingual misoprostol versus standard surgical care for treatment of incomplete abortion in five sub-Saharan African countries

**DOI:** 10.1186/1471-2393-12-127

**Published:** 2012-11-14

**Authors:** Tara Shochet, Ayisha Diop, Alioune Gaye, Madi Nayama, Aissata Bal Sall, Fawole Bukola, Thieba Blandine, Okunlola Michael Abiola, Blami Dao, Ogunbode Olayinka, Beverly Winikoff

**Affiliations:** 1 Gynuity Health Projects, 15 E. 26th St. Suite 801, New York, NY 10010, USA; 2 Centre de Sante le Roi Baudouin, Roi Baudouin Guédiawaye, Senegal; 3 Issaka Gazoby Maternity Hospital, Niamey, Niger; 4 Cheikh Zayed Hospital, Nouakchott, Mauritania; 5 Department of Obstetrics & Gynecology, University College Hospital Ibadan, Oyo State, Nigeria; 6 Centre Hospitalier National Yalgado Ouédraogo, Ouagadougou, Burkina Faso; 7 Centre Hospitalier National Souro Sanou, Bobo Dioulasso, Burkina Faso

**Keywords:** Misoprostol, Manual vacuum aspiration (MVA), Incomplete abortion, Post-abortion care (PAC)

## Abstract

**Background:**

In low-resource settings, where abortion is highly restricted and self-induced abortions are common, access to post-abortion care (PAC) services, especially treatment of incomplete terminations, is a priority. Standard post-abortion care has involved surgical intervention but can be hard to access in these areas. Misoprostol provides an alternative to surgical intervention that could increase access to abortion care. We sought to gather additional evidence regarding the efficacy of 400 mcg of sublingual misoprostol vs. standard surgical care for treatment of incomplete abortion in the environments where need for economical non-surgical treatments may be most useful.

**Methods:**

A total of 860 women received either sublingual misoprostol or standard surgical care for treatment of incomplete abortion in a multi-site randomized trial. Women with confirmed incomplete abortion, defined as past or present history of vaginal bleeding during pregnancy and an open cervical os, were eligible to participate. Participants returned for follow-up one week later to confirm clinical status. If abortion was incomplete at that time, women were offered an additional follow-up visit or immediate surgical evacuation.

**Results:**

Both misoprostol and surgical evacuation are highly effective treatments for incomplete abortion (misoprostol: 94.4%, surgical: 100.0%). Misoprostol treatment resulted in a somewhat lower chance of success than standard surgical practice (RR = 0.90; 95% CI: 0.89-0.92). Both tolerability of side effects and women’s satisfaction were similar in the two study arms.

**Conclusion:**

Misoprostol, much easier to provide than surgery in low-resource environments, can be used safely, successfully, and satisfactorily for treatment of incomplete abortion. Focus should shift to program implementation, including task-shifting the provision of post-abortion care to mid- and low- level providers, training and assurance of drug availability.

**Trial registration:**

This study has been registered at clinicaltrials.gov as NCT00466999 and NCT01539408

## Background

Complications of pregnancy failure (spontaneous or induced abortion) are a major cause of maternal morbidity and mortality, particularly in low-resource settings where abortion is highly restricted and self-induced abortions are common [1]. In these countries access to post-abortion care (PAC) services, especially treatment of incomplete terminations, is a priority.

Standard post-abortion care has involved surgical intervention, such as dilatation and curettage (D&C) or vacuum aspiration (manual or electric), to complete uterine evacuation and prevent infection. However, in low-resource areas, surgical methods can be difficult to access due to a scarcity of trained providers, sterile surgical facilities, and/or inadequate transportation to higher level centers where surgical procedures are most often performed. Misoprostol, a drug which is easy to store and requiring no surgical skills to administer, provides an alternative to surgical intervention that could increase access to abortion care. It will also contribute to task-shifting efforts by enabling lower level providers to manage post-abortion care. This could be particularly helpful in places with limited human resources.

Misoprostol has been established as an effective alternative to surgical care; randomized trials comparing 600 mcg of oral misoprostol to manual vacuum aspiration (MVA) for treatment of incomplete abortion found comparable efficacy (91.0% - 99% for misoprostol vs. 91.5% - 100% for MVA) and equal or higher levels of satisfaction among women assigned to the misoprostol arms [2-5]. An extensive review concluded that 600 mcg of oral misoprostol should be recommended for treatment of incomplete abortion [6]. An additional randomized study established 400 mcg of sublingual misoprostol as equally effective as the 600 mcg oral regimen with similar side effects and participant satisfaction [7]. Another study confirmed similar efficacy rates for 400 mcg sublingual misoprostol and MVA [8]. Using 400 mcg misoprostol instead of 600 mcg might mean significant cost reduction in treatment, particularly important in places where misoprostol is expensive relative to income levels.

We sought to gather additional evidence regarding 400 mcg of sublingual misoprostol vs. standard surgical care for treatment of incomplete abortion in the environments where need for economical non-surgical treatments may be most useful.

## Methods

Data from one multi-site (Mauritania, Niger and Senegal) and two country-level (Burkina Faso and Nigeria) randomized trials comparing sublingual misoprostol to standard surgical care for treatment of incomplete abortion were combined. All women presenting to one of the study sites (located in Guédiawaye, Senegal; Nouakchott, Mauritania; Niamey, Niger; Ibadan, Nigeria; and Ouagadougou and Bobo Dioulasso, Burkina Faso), who lived or worked within 1 hour travel time, who had a confirmed incomplete abortion (spontaneous or induced), and who met the study criteria were invited to participate. An eligible incomplete abortion was defined as past or present history of vaginal bleeding during pregnancy and an open cervical os (if ultrasound not used) or evidence of incomplete abortion with substantial debris in uterus, if ultrasound used. Requirements for eligibility included no contraindications to the study drug, uterine size no larger than that consistent with 12 week gestation at time of presentation for care, no signs of severe infection, no hemodynamic disturbances, general good health, and willingness to provide contact information for purposes of follow-up. Women who were suspected of having an ectopic pregnancy were not eligible for the study. All participants would have been advised to have a surgical evacuation of the uterus if misoprostol were not available. Similar study protocols were approved by ethical review boards in each country^a^ and all participants gave written, informed consent. The trials included in this paper are registered as two separate studies on clinicaltrials.gov (NCT00466999 and NCT01539408).

Participants were randomly assigned to either one dose of 400 mcg of sublingual misoprostol (200 mcg tablet × 2) (Cytotec®, Pfizer, USA) or a surgical evacuation of the uterus following standard practice at each hospital (MVA or D&C). (The multicenter sites were randomized together and the two country-level sites were randomized individually). Women assigned to the misoprostol group took the pills at the hospital and were instructed to hold the pills under their tongues for 30 minutes and then swallow any remaining tablet fragments. They were then discharged at the provider’s discretion. Women assigned to the surgical arm received immediate surgical evacuations at the study hospital. Analgesics and antibiotics for participants receiving surgical treatment were given per each facility’s clinical norms; no anesthesia was used during the procedures.

Participants were asked to return to the clinic 1 week later to confirm clinical status. In the event of continued heavy bleeding, an enlarged uterus, or any suspicion of an ectopic pregnancy, the woman was referred for ultrasound and follow-up care. If continued incomplete abortion was determined by clinical exam or by ultrasound in either of the study arms, women were given the option of an immediate surgical evacuation or returning for additional follow-up 1 week later to see if expulsion had occurred spontaneously. If, after the second follow-up visit, abortion was not complete, a surgical completion was performed. All women were advised that they could return to the study site at any time if complications arose, or if they had questions. Care throughout the study was provided by clinicians at all levels. In all sites except Mauritania and Nigeria, care was provided by midwives or nurses. In both these sites, care was mostly given by physicians although nurses and midwives were involved in counseling and follow-up. Only some clinicians at the Burkina Faso sites had previous experience with misoprostol for treatment of incomplete abortion [4].

A sample size of 390 (195 per arm) was needed to detect a 5% one-sided difference between surgical (assumed to be 98% effective) and sublingual misoprostol with a 95% confidence interval and 80% power. Data on demographics, method outcome, side effects, and women’s satisfaction with the treatment were combined from the 6 sites and analyzed using relative risk, chi-square, and independent t-tests as appropriate. Use of ultrasound and any necessary interventions were also documented and evaluated. Data were entered in SPSS (Version 15.0, Chicago, IL). All analyses were conducted with Stata software (Version 11, College Station, TX).

## Results

**Figure 1:**
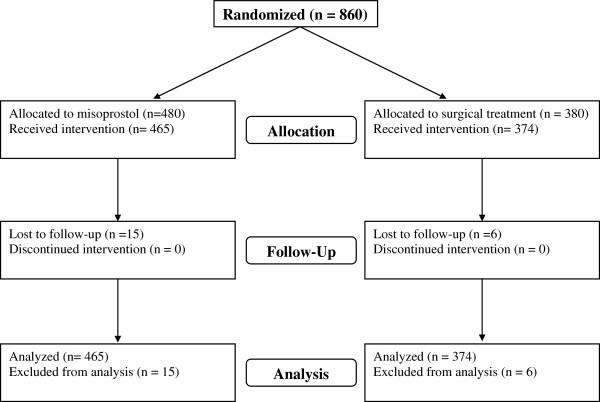
Study flowchart.

A total of 860 women were enrolled in the study between May 2007 and October 2010 (Figure 1). Participants were randomly assigned to 1 of the 2 study groups: 480 to misoprostol and 380 to standard surgical treatment. (In Burkina Faso, the misoprostol arm was oversampled with a 2:1 allocation to gain additional experience in using this method). Twenty-one women were lost to follow-up before study completion and therefore are only included in the demographic analyses. We examined participant characteristics including age, parity, education, marital status, and abortion type (spontaneous or induced) (Table 1).

**Table 1:** **Participants**’ characteristics*

	**Group 1:Misoprostol**	**Group 2:Surgical**	**p-value**
	**n = 480**	**n = 380**	
Age in years: mean ± SD (range)	28.1 ± 7.2 (13-48)^a^	28.7 ± 7.3 (14-46)^b^	0.19
Education level: % (n)			0.63
No education	30.6 (118/385)	28.3 (80/283)	
Primary	24.9 (96/385)	23.7 (67/283)	
Secondary	30.9 (119/385)	31.1 (88/283)	
Tertiary	13.5 (52/385)	17.0 (48/283)	
Marital status: % (n)			0.20
Single	11.5 (55/478)	8.3 (31/372)	
Married	87.0 (416/478)	90.9 (338/372)	
Divorced	1.5 (7/478)	0.8 (3/372)	
Parity: mean ± SD (range)	2.1 ± 2.1 (0-11)^c^	2.3 ± 2.2 (0-11)^d^	0.09
Abortion type according to provider: % (n)			0.23
Spontaneous	94.0 (440/468)	95.9 (349/364)	
Induced	6.0 (28/468)	4.1 (15/364)	

^*^ Denominators vary due to response rate for each question.

^a^ n = 478.

^b^ n = 375.

^c^ n = 479.

^d^ n = 377.

Treatment of incomplete abortion with both methods resulted in complete uterine evacuation for more than 9 out of 10 women. Efficacy with the misoprostol was slightly lower: 94.4% of the women who received misoprostol and 100% of the women who received surgical intervention had successful evacuations (RR = 0.90; 95% CI: 0.88-0.92) (Table 2). The majority of the successful evacuations were confirmed at the first follow-up (91.8% (393/428) in the misoprostol arm and 99.4% (360/362) in the surgical arm; data not shown). There was variation in success with misoprostol by site, with completion rates ranging from 88.7% in Niger to 97.6% in Burkina Faso, where providers had previous experience using misoprostol for treatment of incomplete abortion. At the beginning of the study at three of the sites (Senegal, Niger, and Mauritania), many failures with misoprostol (the new treatment) were recorded; success rates increased considerably after the first month of misoprostol exposure as providers became more familiar with the method. At these sites, complete uterine evacuation was recorded for 76.2-86.2% of participants receiving misoprostol in the first month of recruitment and increased to 92.3-95.7% during the remaining months (data not shown). In Niger, all but 1 failure occurred in the first 3 months of recruitment (data not shown). And in Senegal, all but 1 failure occurred in the first 5 weeks of recruitment (data not shown).

**Table 2:** Results

	**Misoprostol**	**Surgical**	**RR(95% CI) / p-value**
	**n = 465**	**n = 374**	
Success: % (n)^a^			
Complete abortion	94.4 (439)	100.0 (374)	0.90 (0.88-0.92)
Incomplete abortion/ongoing pregnancy	5.6 (26)	0.0 (0)	
Success by site			
Senegal	92.9 (92/99)	100.0 (100/100)	0.89 (0.84-0.94)
Niger	88.7 (63/71)	100.0 (81/81)	0.85 (0.78-0.92)
Mauritania	91.4 (53/58)	100.0 (61/61)	0.87 (0.81-0.94)
Nigeria	96.2 (25/26)	100.0 (25/25)	0.92 (0.85-0.99)
Burkina Faso	97.6 (206/211)	100.0 (107/107)	0.97 (0.97-0.97)
Ultrasound used to evaluate success^b^: % (n)	39.1 (167/427)	17.8 (60/338)	p < 0.001
Senegal^c^	81.1 (77/95)	18.6 (16/86)	p < 0.001
Other 4 sites	27.1 (90/332)	17.5 (44/252)	p < 0.01
Women who made an unscheduled visit^d^	2.9 (13/452)	1.1 (4/366)	p = 0.14
Women who called the provider^e^	6.0 (27/448)	1.1 (4/364)	p < 0.01

^a^21 women from the Niger site were lost to follow-up: 15 in the misoprostol arm and 6 in the surgical arm.

^b^Ultrasound data is not available for 74 women: 38 in the misoprostol arm and 36 in the surgical arm.

^c^Senegal was required by their IRB to use ultrasound in evaluating outcome.

^d^3 women in the misoprostol arm made 2 unscheduled visits; all others made only 1 unscheduled visit.

^e^4 women in the misoprostol arm called twice; all other callers called once.

Use of ultrasound to determine outcome was more likely to occur with women in the misoprostol arm than with those in the surgical arm (Table 2). In Senegal, ultrasound use for misoprostol participants was required by the local ethics committee and was used in over 81% of the cases to confirm outcome. (Use decreased from 90% in the first half to 52% in the second half of the study). Among the other 4 sites, ultrasound use was also significantly higher in the misoprostol group (27.1% vs. 17.5%, p < 0.01).

Severity of side effects was similar for participants in the 2 study arms (Table 3). Slightly more women in the misoprostol group than in the surgical group reported that side effects were tolerable or easily tolerable (misoprostol: 77.5%, surgical: 71.0%; p = 0.02); however, very few women in either study group reported side effects as severe or very severe (misoprostol: 7.7%, surgical: 5.0%). Although reports of pain and/or cramps were significantly greater in the misoprostol group (54.8% vs. 24.4%, p < 0.001), reports of tolerability of pain were greater in that group as well with 92.2% of the misoprostol group and 85.3% of the surgical group reporting that pain was “tolerable” or “very tolerable” (p < 0.01). Finally, where reported, almost all women in both groups reported that treatment was either “a little difficult” or “not at all difficult” and that the bleeding was “acceptable” or “very acceptable.”

**Table 3:** **Side**-**effects**: % (**n**)*

	**Misoprostol**	**Surgical**	**p-value**
	**n = 465**	**n = 374**	
Severity of side effects			
“None”	14.8 (65/440)	24.0 (53/221)	0.02
“Easily tolerable” / “tolerable”	77.5 (341/440)	71.0 (157/221)	
“Severe” / “very severe”	7.7 (34/440)	5.0 (11/221)	
Normal bleeding**	35.4 (119/336)	9.4 (17/180)	< 0.001
Heavy bleeding**	11.6 (39/336)	0.6 (1/180)	< 0.001
Spotting***	31.0 (104/336)	51.1 (92/180)	< 0.001
Pain/cramps**	54.8 (184/336)	24.4 (44/180)	< 0.001
Fever/chills**	3.9 (13/336)	1.7 (3/180)	0.20
Nausea**	16.7 (56/336)	2.8 (5/180)	< 0.001
Vomiting**	7.1 (24/336)	1.1 (2/180)	< 0.01
Headache^†^	7.7 (26/336)	8.9 (16/180)	0.74
Gastrointestinal issues^†^	15.2 (51/336)	3.9 (7/180)	< 0.001
Vertigo^†^	1.2 (4/336)	4.4 (8/180)	0.03
Tolerability of pain**			
“No pain”	5.1 (17/332)	5.6 (10/177)	< 0.01
“Very tolerable” / “tolerable”	92.2 (306/332)	85.3 (151/177)	
“Not tolerable”	2.7 (9/332)	9.0 (16/177)	
Difficulty of treatment***			0.45
“Not at all difficult” / “a little difficult”	95.4 (290/304)	97.4 (148/152)	
“Difficult” / “very difficult”	4.6 (14/304)	2.6 (4/152)	
Acceptability of bleeding**			
“Acceptable” / “very acceptable”	99.1 (328/331)	100.0 (174/174)	0.56
“Not acceptable”	0.9 (3/331)	0.0 (0/0)	

^*^Denominators vary due to response rate for each question.

**Data only collected in Senegal, Nigeria, and Burkina Faso.

***Data only collected in Senegal and Burkina Faso.

^†^Listed in “other” side effects; only collected in Senegal, Nigeria, and Burkina Faso.

Satisfaction with the procedure was very high in both study groups, with almost all women “satisfied” or “very satisfied” (Table 4). However, significantly more women in the misoprostol than in the surgical arm would select their method again (97.6% vs. 87.8%, p < 0.001) and would recommend their method to a friend (97.6% vs. 85.8%, p < 0.001).

**Table 4:** **Overall acceptability of treatment**: % (**n**)*

	**Misoprostol**	**Surgical**	**p-value**
	**n = 465**	**n = 374**	
Satisfaction with procedure			
“Satisfactory” / “very satisfactory”	98.5 (452/459)	98.1 (314/320)	0.78
“Unsatisfactory” / “very unsatisfactory”	1.5 (7/459)	1.9 (6/320)	
Would select this method again, if needed	97.6 (448/459)	87.8 (274/312)	< 0.001
Would recommend this method to a friend	97.6 (448/459)	85.8 (271/316)	< 0.001

^*^Denominators vary due to response rate for each question.

## Discussion

These data present yet another example of how misoprostol can be successfully incorporated into standard post-abortion care services [2-4,6,7]. The difference in side effects between the two groups is also comparable to what has been shown in similar studies [4,5,8]. Of note, the success rates for misoprostol at three of the sites were much higher after the first month of use. This “learning curve” for the clinicians offering misoprostol provides an excellent example of what may occur when misoprostol is first made available as a method of uterine evacuation in new settings.

The majority of surgical interventions in our study were initiated by women’s concerns over the amount and/or duration of bleeding; it is certainly possible that at least some of the interventions would not have been carried out by a more experienced misoprostol provider. Given that most interventions were carried out in the early stages of the study, providers may also have been less comfortable with the new method. And providers’ lack of comfort may have translated to women’s unease with the process. Indeed, it is not uncommon to see success rates improve over time when misoprostol is first introduced; providers who have not previously used misoprostol for treating incomplete abortion (or for medical abortion) need to become accustomed to the process, particularly the bleeding patterns, in order not to intervene prematurely. For example, in a recent study of misoprostol for incomplete abortion in Ghana [9], all failures occurred in the first half of the study (J. Taylor, personal communication, September 20, 2011). In Turkey, a study with a low rate of success with medical abortion was followed by a second study with a much higher completion rate after more extensive provider education and experience were provided [10,11]. And in our study, Burkina Faso had the highest success rate both at the beginning of the study and overall, and was the only country to have previous experience with the method. This pattern suggests that when introducing misoprostol into new settings, additional focus should be placed on provider training as well as on provider follow-up and support to ensure that clinicians are fully comfortable with both the amount and duration of side effects, especially bleeding.

As in many studies addressing abortion and/or post-abortion care in settings with limited legal access to safe abortion care, some women were lost to follow-up. High rates of loss to follow-up are common with incomplete abortion treatment, particularly where cost and distance to care are substantial factors, as well as in settings where abortion is illegal [2,5]. Moreover, cultural norms in some settings discourage seeking care when it is not necessary, suggesting that women with complications are more likely to return and those not returning are more likely to have had no further problems [5]. One proposed solution to poor in-clinic follow-up is to develop an at-home assessment tool with a symptom checklist to help women determine whether or not they need to return for further care [2]. Telephone follow-up could also be established to reduce the need for in-person assessment [12].

While the sites in this study were all secondary and tertiary level hospitals, the findings suggest a great potential for use of misoprostol at lower-level facilities where physicians are less available. As demonstrated in 4 of the 6 sites, physicians are not needed to provide PAC services: both counseling and provision of the medications were successfully provided by other clinicians such as nurses or midwives trained in treatment protocols. However, more attention to training in how to avoid unnecessary intervention would be beneficial. The option of a first line treatment at out-patient clinics could reduce the burden on higher-level settings where case loads are high and also increase access for women who may have difficulties reaching higher level care. This would facilitate task shifting and reduce the human resource burden in many countries.

This study supports previous research showing that ultrasound is not necessary for outcome assessment when treating incomplete abortion with misoprostol [13-15]. The majority of misoprostol cases were successfully evaluated without the use of ultrasound. And in Senegal, where ultrasound use was required, its use decreased substantially over the course of the study (as providers likely became more comfortable with the process), and clinicians relied more on clinical assessment alone. Misoprostol may be very appropriate in places where ultrasound is not available and/or where providers are not trained in ultrasound use.

There were limitations to this study including small sample size across different sites, the mandated use of ultrasound in Senegal, and the differences in standard of care at each location. However, regardless of these limitations, this research gives us a good overview of what service provision of misoprostol for treatment of incomplete abortion might be like in these settings.

## Conclusion

The evidence is substantial and quite clear that misoprostol can be used safely, successfully, and satisfactorily for treatment of incomplete abortion. This observation is particularly important for locations where surgical interventions are less available and/or less acceptable. During the course of this study, the World Health Organization added misoprostol for incomplete abortion to their essential medicines list (EML) [16]. With the established success of both oral and sublingual routes, there are two viable misoprostol options for PAC services. In low-resource settings these protocols are critical for moving forward. Focus now needs to shift to program development and implementation, including more extensive provider training and assurance of drug availability. Programs created to translate the research into practice are critical to realize the potential for this technology to improve women’s health.

## Endnotes

^a^Mauritania: The Allendale Investigational Review Board; Niger: Ministry of Health Ethical Review Board; Senegal: National Counsel on Health Research, National Ethical Committee, Ministry of Health and Prevention; Burkina Faso: Burkina Faso Ministry of Health Ethical Review Board; Nigeria: Ethical Committee of the College of Medicine and the University of Ibadan/University College Hospital.

## Competing interests

The authors declare that they have no competing interest.

## Authors’ contributions

TS: conducted data analysis, data interpretation and wrote draft of manuscript. AD: participated in the design of the study, conducted data analysis, data interpretation and participated in the writing of manuscript. AG, MN, FB, ABS, TB, BD, OMA, OO: provided patient treatment, participated in data collection and interpretation. BW conceived of the study, participated in its design and helped draft the manuscript. All authors read and approved the final manuscript.

## Pre-publication history

The pre-publication history for this paper can be accessed here:

http://www.biomedcentral.com/1471-2393/12/127/prepub
